# COPD: pulmonary vascular volume associated with cardiac structure and function

**DOI:** 10.1007/s10554-023-03027-1

**Published:** 2023-12-01

**Authors:** Lisa Steen Duus, Ditte Vesterlev, Anne Bjerg Nielsen, Mats Højbjerg Lassen, Pradeesh Sivapalan, Charlotte Suppli Ulrik, Therese Lapperre, Andrea Browatzki, Rubén San José Estépar, Pietro Nardelli, Jens-Ulrik Staehr Jensen, Raúl San José Estépar, Tor Biering-Sørensen

**Affiliations:** 1 Dept. of Cardiology, Herlev & Gentofte Hospital, University of Copenhagen, Copenhagen, Denmark; 2 Applied Chest Imaging Laboratory, Dept. of Radiology, Applied Chest Imaging Laboratory, Brigham and Women’s Hospital, Harvard Medical School, Boston, USA; 3 Depart. of Internal Medicine, Respiratory Medicine Section, Herlev and Gentofte Hospital, Herlev and Gentofte, Denmark; 4 Depart. of Respiratory Medicine, Copenhagen University Hospital – Hvidovre, Hvidovre, Denmark; 5 Depart. of Respiratory Medicine, Copenhagen University Hospital – Bispebjerg, Copenhagen, Denmark; 6 Depart. Of Respiratory Medicine, Antwerp University Hospital, Antwerp, Belgium; 7 Laboratory of Experimental Medicine and Pediatrics, University of Antwerp, Antwerp, Belgium; 8 Depart. of Respiratory and Infectious Diseases, North Zealand Hospital, Frederikssund and Hilleroed, Denmark; 9 Dept. of Biomedical Sciences, Faculty of Health and Medical Sciences, University of Copenhagen, Copenhagen, Denmark

**Keywords:** Artificial intelligence, Cardiac structure and function: computed tomography, COPD, Echocardiography

## Abstract

**Background:**

Early recognition of cardiac dysfunction in patients with chronic obstructive pulmonary disease (COPD) may prevent future cardiac impairment and improve prognosis. Quantitative assessment of subsegmental and segmental vessel volume by Computed Tomographic (CT) imaging can provide a surrogate of pulmonary vascular remodeling. We aimed to examine the relationship between lung segmental- and subsegmental vessel volume, and echocardiographic measures of cardiac structure and function in patients with COPD.

**Methods:**

We studied 205 participants with COPD, included in a large cohort study of cardiovascular disease in COPD patients. Participants had an available CT scan and echocardiogram. Artificial intelligence (AI) algorithms calculated the subsegmental vessel fraction as the vascular volume in vessels below 10 mm^2^ in cross-sectional area, indexed to total intrapulmonary vessel volume. Linear regressions were conducted, and standardized ß-coefficients were calculated. Scatterplots were created to visualize the continuous correlations between the vessel fractions and echocardiographic parameters.

**Results:**

We found that lower subsegmental vessel fraction and higher segmental vessel volume were correlated with higher left ventricular (LV) mass, LV diastolic dysfunction, and inferior vena cava (IVC) dilatation. Subsegmental vessel fraction was correlated with right ventricular (RV) remodeling, while segmental vessel fraction was correlated with higher pulmonary pressure. Measures of LV mass and right atrial pressure displayed the strongest correlations with pulmonary vasculature measures.

**Conclusion:**

Pulmonary vascular remodeling in patients with COPD, may negatively affect cardiac structure and function. AI-identified remodeling in pulmonary vasculature may provide a tool for early identification of COPD patients at higher risk for cardiac impairment.

## Introduction

Chronic obstructive pulmonary disease (COPD) is one of the leading causes of morbidity and mortality worldwide. COPD is often associated with cardiovascular diseases (CVD) and cardiac impairment [1, 2]. The presence of cardiovascular comorbidities is associated with an increased risk for hospitalization and longer length of stay [3]. Recognition of early signs of cardiac dysfunction could identify COPD patients at high risk of future cardiac impairment and by that improve the long-term outcome.

Assessment of pulmonary vascular abnormalities with artificial intelligence (AI) using neural networks [4–6], has generated new possibilities for understanding and quantifying vascular abnormalities using Computed Tomographic (CT) imaging. Radiographic abnormalities of the pulmonary vessels, such as lower total pulmonary vascular volume (TPVV) and relatively less small - and subsegmental vessel blood volume (i.e., more pruning) are commonly seen across several pulmonary diseases, including COPD [7]. Radiographic pruning, described as vascular remodeling and loss of the small pulmonary vessels, has previously been associated with the severity of lung disease, including the extent of emphysema, lower level of lung function, more severe pulmonary hypertension, and increased mortality among individuals with COPD [7]. Moreover, radiographic pruning has been associated with cardiac disease in terms of impaired left ventricular filling and right ventricular (RV) dysfunction [2]. A previous study has found an association between pruning and RV epicardial volume assessed by CT imaging [8]. However, the association between subsegmental vasculature and central segmental, and cardiac function has not previously been explored.

We aimed to investigate total pulmonary vessel volume, subsegmental vessel volume fraction and segmental vessel volume fraction, and its association with cardiac structure and function as assessed by conventional and novel echocardiographic measurements.

## Methods

### Patient population

A subset of 244 participants suffering from chronic obstructive pulmonary disease (COPD) were included from the Copenhagen COPD and Echo study, a larger ongoing prospective cohort study. All participants signed an informed consent form prior to participation. The project is approved by the Danish data protection agency and Danish Research Ethics Committee and complies with the 2nd Declaration of Helsinki.

All participants had an echocardiogram performed and blood samples taken at the date of inclusion. Participants were included in the current sub-study if they had a specialist verified COPD diagnosis and available CT scan within 5 years prior to the inclusion (n = 244). CT scans with a section thickness of 1–3 mm were used. Patients with CT scans not fitting the section thickness criteria, were excluded (n = 14). Furthermore, we excluded patients where the CT analysis failed during the computational vascular reconstruction process (n = 18), the vascular reconstruction failed the manual quality check (n = 5) (explained below in the CT methods section), and patients who did not have an available echocardiogram at the time of analysis (n = 2). Hence, the final study population included 205 patients.

Demographics, clinical and laboratory data were registered from questionnaires and electronic medical records.

Specialist-verified COPD was defined and staged by the Global Initiative for Chronic Obstructive Lung Disease (GOLD) criteria [9] for disease severity stages 1, 2, 3, and 4. Spirometry test results were collected from medical records and expected lung function values were calculated automatically using existing guidelines [10]. Hypertension was defined as systolic blood pressure above 140 mmHg, diastolic blood pressure above 90 mmHg, self-reported hypertension, or the use of antihypertensive medication. Diabetes was defined as HbA1c ≥ 48 mmol/mol, self-reported diabetes, or the use of antidiabetic medicine.

### Echocardiography

All echocardiographic examinations were performed using Vivid 9 ultrasound machines (GE Healthcare, Norway). Echocardiograms were stored digitally in a remote archive and analyzed offline with available software (BT2.02 EchoPac, GE Healthcare, Norway). All echocardiographic analyses were performed by a single investigator blinded to clinical characteristics and outcomes.

Measurements of the LV dimensions (interventricular septal diameter in diastole (IVSd) and diastolic LV posterior wall thickness (LVPWD)) were measured in the parasternal long-axis view.

The LV mass index (LVMI) was calculated from these linear measurements. LV ejection fraction (LVEF) was calculated using the modified Simpson’s biplane method from the apical 4- and 2-chamber views. IVC diameter was measured in the subcostal view. Mitral inflow velocities (E, A, E/A, E-wave deceleration time) were assessed using pulsed-wave Doppler from the apical 4-chamber view at the level of the mitral valve leaflet tips. Pulsed-wave tissue Doppler imaging was used to measure early relaxation velocity (e´). The LA end-systolic volume was measured by the biplane area-length method and indexed to body surface area (BSA) to acquire the LA volume index (LAVI). Tricuspid annular systolic plane excursion (TAPSE) was measured by M-mode as the longitudinal displacement of the lateral tricuspid annulus during systole. RV end-diastolic and end-systolic areas were measured in the apical view by tracing the RV endocardium in systole and diastole. Fractional area change (FAC) was calculated as the difference between RV end-diastolic area and the end-systolic area. The tricuspid regurgitation (TR) peak velocity was measured as the peak velocity of the TR jet using CW Doppler, identified either in the parasternal short-axis view or apical 4-chamber view with the help of color Doppler. TR maximal pressure gradient (TR max PG) was calculated from the pulmonary regurgitation velocity. Speckle tracking echocardiography of the LV was performed in the apical 4-chamber, 2-chamber, and apical long-axis view to acquire the global longitudinal strain (GLS). Analyses were performed by placing two points at the basal level and one point at the apex after which the automated software generated a region of interest that covered the LV wall. Segments with persistent inadequate tracking were excluded from analysis and GLS was calculated by averaging values measured in the remaining segments. RV speckle tracking was performed in the apical 4-chamber view by manual delineation of the endocardial border of the RV myocardial wall [11]. RV free wall longitudinal strain (RVFWS) was calculated as the mean of the RV lateral wall segments.

### CT-derived pulmonary vessel volume

**Fig. 1:**
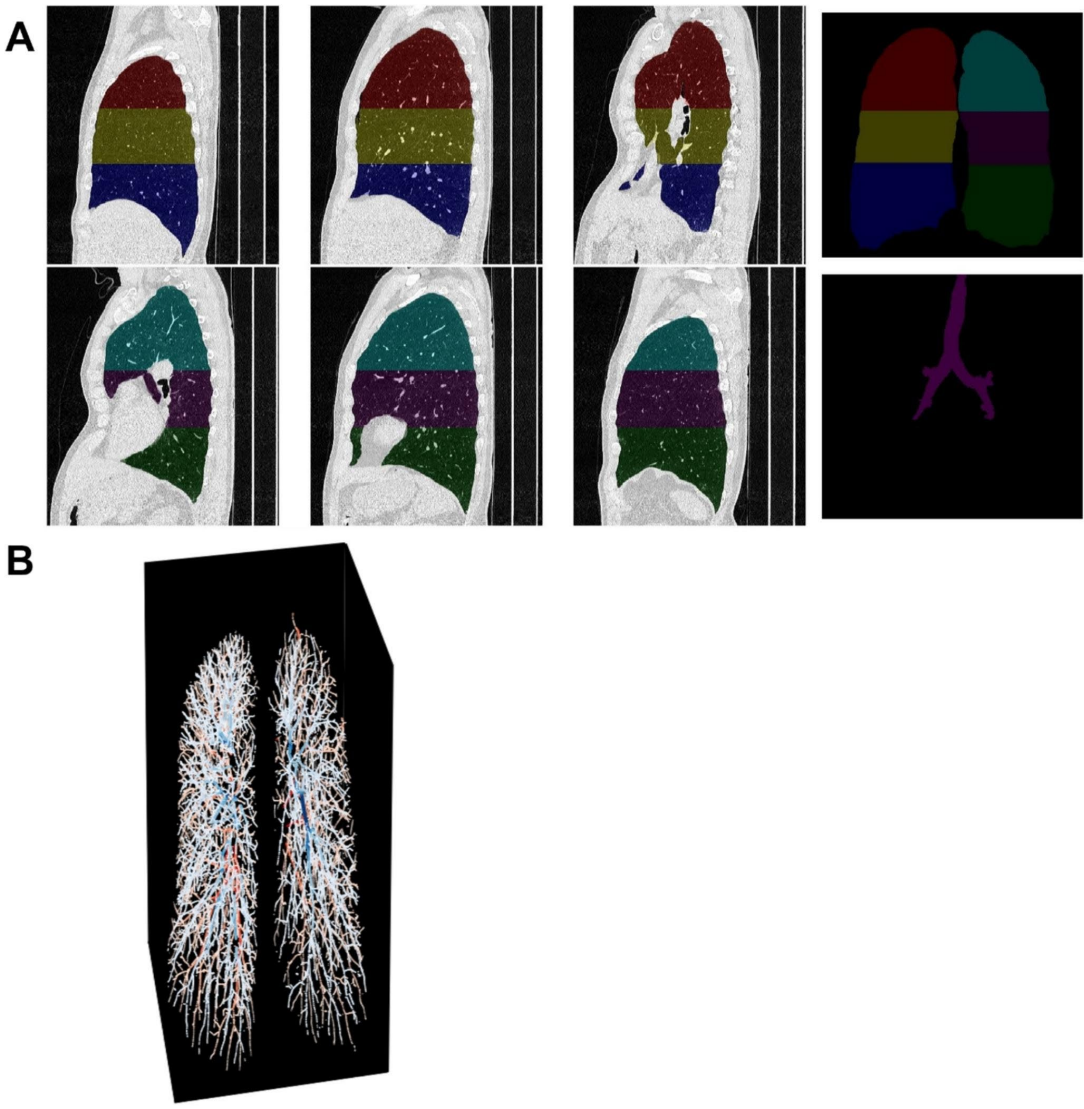
An example of vascular reconstruction quality check outputs **A)** depicts the output from the partial quality check. This step is used to make sure that the region of interest only includes the lung, and other organs i.e., the heart **B)** depicts the output from the total vascular reconstruction. The quality check is used to make sure that larger regions of the pulmonary vasculature are not missing

Clinical CT examinations covering the entire thorax were acquired from the electronic medical record. CT scans with a section thickness of 1–3 mm were used. It was independent of this study whether the CT was performed with contrast fluid or without. Image analysis was performed in collaboration with the Applied Chest Imaging Laboratory at Brigham and Women’s Hospital, Harvard Medical School, utilizing automated software based on the Chest Imaging Platform (www.chestimagingplatform.org) as previously described [4]. Lung field masks were extracted using a region-growing approach [12]. The pulmonary vasculature was extracted using a validated scale-space particle approach [13, 14] and the vessel scale was used to determine the vessel caliber [4]. After the automated CT analysis, the processing pipeline was manually verified. This included verification of lung masks to fit within the CT lung region and exclusion of CT scans with artifacts (Fig. 1). After the automated CT analysis, the vascular extraction was visually verified (Fig. 1). The following were calculated; the volume of vessels of varying sizes, measured as cross-sectional area, (Fig. 1); the total pulmonary vessel volume (TPVV), the subsegmental vessel volume, and the segmental vessel volume. The TPVV was measured to include both vessel walls and luminal blood. The TPVV included the combined intraparenchymal pulmonary vessels, while the blood volume in the subsegmental vessel was defined as vessels with nominal sizes below 10 mm^2^ in cross-sectional area (CSA) (BV < 10) and the segmental vessel in vessels within a nominal size of 20 to 90 mm^2^ in cross-sectional area (BV20-90).

To account for inherent anatomic variability in blood vessel volumes (e.g., height and body mass index (BMI)), the vessel volumes mentioned above were indexed to the total pulmonary vessel volume TPVV. The indexed measurements reflect how big a fraction of the TPVV is composed of respectively subsegmental vessels (the subsegmental vessel fraction; BV < 10 indexed to TPVV) and segmental vessels (the segmental vessel fraction; BV 20–90 indexed to the TPVV).

The subsegmental vessel fraction, representing the relative distribution of blood vessel volume in the smallest peripheral blood vessel detectable by CT, was utilized as a measure reflecting both pulmonary vascular pruning, defined as a lower quantity of peripheral pulmonary vessels, and vasoconstriction [7]. The segmental vessel fraction represents the fraction of blood volume in the larger vessel. A higher segmental vessel fraction indicates a shift of blood volume from the subsegmental vessels to the segmental vessels, which is believed to be a compensatory mechanism for the lower amount of smaller subsegmental vessels.

Emphysema was defined by the measurement of a CT attenuation value of less than − 950 Hounsfield units (LAA-950) [15]. One operator measured the pulmonary artery (PA) and aorta (A) ratio (PA: A ratio) at the bifurcation of the PA using the semiautomatic image-processing program Chest Imaging Platform extension in Slicer 4.10.2 [16].

### Statistical analysis

Baseline characteristics were stratified by subsegmental vessel fraction and divided into tertiles of low, mid, and high subsegmental vessel fraction (high to low; less pruning to more severe pruning). Continuous variables with Gaussian distribution were compared using Student´s T-test and with Mann-Whitney U test if non-Gaussian distributed. Categorical variables were compared using Pearson’s Chi^2^ test. The distribution of data was tested for normality with histograms. Data were expressed as mean ± standard deviation (SD) or median with interquartile range (IQR), as appropriate.

Univariable linear regression was conducted, and standardized ß-coefficients were calculated. Multivariable linear regression was conducted, adjusted for sex, age, degree of emphysema, diabetes mellitus, hypertension, smoking status, ischemic heart disease (defined as prior acute myocardial infarction with or without prior revascularization therapy, or heart failure*)* and time between CT scan and echocardiogram. Scatterplots were created to visualize the continuous correlation between subsegmental vessel fraction and the significantly correlated echocardiographic parameters from the linear regression model. A P-value of ≤ 0.05 was considered statistically significant. All statistical analyses were performed with STATA Statistics, SE 15.1 (StataCorp, College Station, TX, USA).

## Results

Baseline clinical and echocardiographic characteristics, stratified by subsegmental vessel fraction tertiles are shown in Table 1. Participants in the lowest tertile represent the group with the most extensive pruning, while the highest tertile represents the group with the least extensive pruning.

**Table 1:** Baseline characteristics stratified by subsegmental vessel fraction

Factor	Subsegmental vessel fraction Lowest tertile (n = 69) (0.36–0.54)	Subsegmental vessel fraction Mid tertile (n = 68) (0.55–0.61)	Subsegmental vessel fraction Highest tertile (n = 68) (0.62–0.85)	P-value
Age, years	73 ± 7	71 ± 8	70 ± 10	0.22
Male sex, n (%)	51 (74)	38 (56)	25 (37)	0.001
FEV1% predicted, %	49.9 ± 18.6	48.1 ± 15.8	46.8 ± 19.7	0.60
FEV1/FVC	0.53 ± 0.14	0.49 ± 0.13	0.53 ± 0.14	0.26
BMI, kg/m^2^	28 ± 6	27 ± 5	25 ± 5	0.001
Hypertension, n (%)	43 (62)	27 (40)	25 (3)	0.005
Smoking, n (%) Current Former Never	5 (7) 60 (87) 4 (6)	5(7) 58 (85) 5 (7)	11 (16) 49 (72) 8 (12)	0.19
Ischemic heart disease, n (%)	13 (19)	12 (18)	8 (12)	*0.48*
Diabetes, n (%)	17 (25)	7 (10)	5 (7)	0.008
GOLD, n (%) A B C D	10 (15) 32 (48) 2 (3) 23 (34)	8 (13) 28 (46) 3 (5) 22 (36)	11 (17) 30 (48) 3 (5) 19 (30)	0.98
**Biochemical**				
Creatinine, µmol/L	75 (66, 90)	78.5 (69, 90)	69 (62, 77)	0.002
ProBNP, pg/mL	193 (103;587)	163 (94; 299)	218 (96; 381)	0.37
Troponin I above 6, n (%)	46 (72)	42 (69)	41 (66)	0.78
**CT measures**				
PA:A ratio	0.83 ± 0.13	0.79 ± 0.12	0.79 ± 0.11	0.083
Percentage area of emphysema (%LAA-950)	8.07 (1.56; 17.95)	6.69 (1.84; 16.87)	11.78 (2.95; 23.01)	0.26
**Echocardiography**				
LVMI, g/m2	90 (74; 102)	75 (64; 93)	72 (60; 90)	0.005
LVPWD, cm	1.11 (0.22)	1.04 (0.19)	1.01 (0.19)	< 0.001
IVSd, cm	1.18 (0.25)	1.04 (0.21)	1.00 (0.19)	< 0.001
LVEDVi, mL/m^2^	35.8 (27.8; 42.9)	31.3 (26.4; 37.1)	29.8 (25.1; 37.8)	0.013
LVEF, %	55 ± 8	57 ± 9	56 ± 8	0.37
GLS, %	-14 (-17; -12)	-16 (-18; -14)	-16 (-17; -14)	0.069
LAVI, mL/m2	22 (15; 27)	18 (14; 23)	18 (13; 23)	0.037
E/e’	6.6 (5.8; 8.5)	7.2 (6.0; 8.3)	7.5 (6.2; 9.0)	0.13
e’, cm/s	0.08 (0.02)	0.08 (0.02)	0.08 (0.02)	0.88
A, cm/s	0.73 (0.63; 0.84)	0.76 (0.66; 0.84)	0.74 (0.65; 0.89)	0.66
E, cm/s	0.58 (0.47; 0.68)	0.55 (0.48; 0.65)	0.63 (0.54; 0.72)	0.017
E/A	0.76 (0.65; 0.92)	0.75 (0.65; 0.90)	0.84 (0.69; 0.99)	0.031
IVC, mm	19 (5)	17 (4)	16 (5)	0.007
TAPSE, cm	2.23 (0.41)	2.11 (0.35)	2.13 (0.33)	0.12
RVFAC	1.65 (1.57; 1.73)	1.66 (1.59; 1.72)	1.64 (1.56, 1.71)	0.77
RV free wall strain, %	-14.6(-18.6; 12.9)	-15.6 (-18.2; 12.3)	-17.125 (-19.6; -15.2)	0.005
TR max PG, mmHg	27.6 (22.2; 32.7)	24.9 (21.1; 30.0)	24.1 (21.5; 29.0)	0.15

BMI body mass index, e´ early diastolic relaxation velocity, E/A ratio of peak velocity in early diastole to peak velocity in late diastole, E/e´ ratio between early mitral inflow velocity and early diastolic relaxation velocity, endo endocardial, epi epicardial, FEV1 forced expiratory volume in the first second, FEV1/FVC forced expiratory volume indexed to forced vital capacity, GLS global longitudinal strain, IVC inferior vena cava, IVSd interventricular septal thickness in diastole, LAVI left atrial volume index, LVEDVi left ventricular end-diastolic volume index, LVEF left ventricular ejection fraction, LVMI left ventricular mass index, mid myocardial, LVPWD left ventricular posterior wall dimensions, PA:A ratio pulmonary artery to aorta diameter ratio, RV FAC right ventricular fractional area change, RVFWS right ventricular free wall longitudinal strain, TAPSE tricuspid annular plane systolic excursion, TR max PG pressure gradient from the peak tricuspid regurgitation velocity. %LAA-950 = the percentage of low attenuation area less than − 950 HU (defined as emphysema)

The CT scans were performed at a median of 29 months (IQR= [7.8; 32.3] months) before the echocardiogram. Among the 205 participants, 56% were men, the mean age was 71 years, 46% had hypertension, the mean BMI was 26 kg/m2, 10% were current smokers, 82% were former smokers and 16% had previously diagnosed ischemic heart disease. The mean forced expiratory volume in 1. second (FEV1) was 48%. The mean percentage of lung emphysema was 8%, as measured by LAA-950.

There were significant differences in the clinical characteristics of sex, BMI, hypertension, diabetes, and creatinine between the tertiles of subsegmental vessel fraction. Participants in the lowest subsegmental vessel fraction tertile were more often men, had higher BMI and had more frequent diabetes and hypertension compared to the mid and high subsegmental vessel fraction tertiles.

Regarding cardiac structure and function, patients with the lowest tertile of subsegmental vessel fraction and thus the most extensive degree of pruning, had, compared to the other two tertiles, more pronounced LV thickening with a higher LVMI, LVPWD, and IVSd (Table 1). Furthermore, there was a significant difference in LVEDVi among the subsegmental vessel fraction tertiles. The lowest tertile of low subsegmental vessel fraction had a greater IVC diameter and a higher LAVI (Table 1).

The continuous correlations between TPVV, subsegmental and segmental vessel fractions and measures of cardiac structure and function are shown in Tables 2 and 3. In the unadjusted analysis, we found that TPVV had a significant positive correlation with LVMI, LVPWD, IVSd and LVEDVi. In the unadjusted analysis, we found that the subsegmental vessel fraction had a statistically significant positive correlation with E/e´, and a negative correlation with LVMI, LVPWD, IVSd, LVEDVi, IVC, RVFWS, and TAPSE. Similar correlations were found for segmental vessel fraction, where segmental vessel fraction was positively correlated with LVMI, LVPWD, IVSd, LVEDVi, IVC, TAPSE, and TR max PG.

**Table 2:** Univariable linear regression models for subsegmental and segmental vessel fraction

	Total pulmonary vessel volume		Subsegmental vessel fraction		Segmental vessel fraction	
*Variable*	*Standardized Beta (± SD)*	*P-value*	*Standardized Beta (± SD)*	*P-value*	*Standardized Beta (± SD)*	*P-value*
LVMI	0.242	***0.004***	-0.309	***< 0.001***	0.326	***< 0.001***
LVPWD	0.309	***< 0.001***	-0.278	***0.001***	0.303	***< 0.001***
IVSd	0.271	***0.001***	-0.369	***< 0.001***	0.397	***< 0.001***
LVEDVi	0.272	***< 0.001***	-0.234	***0.001***	0.225	***0.001***
LVEF	-0.092	*0.228*	-0.010	*0.892*	0.029	*0.708*
GLS	0.123	*0.862*	-0.093	*0.187*	0.095	*0.178*
E/A	0.046	*0.519*	-0.008	*0.912*	-0.002	*0.978*
E/e´	-0.100	*0.165*	0.157	***0.029***	-0.124	*0.083*
IVC	0.148	*0.084*	-0.301	***< 0.001***	0.250	***0.003***
TAPSE	0.075	*0.290*	-0.140	***0.048***	0.082	*0.246*
RVFWS	0.004	*0.964*	-0.158	***0.040***	0.132	*0.087*
TRmax PG	0.071	*0.405*	-0.144	*0.089*	0.234	***0.005***

E/A ratio of peak velocity in early diastole to peak velocity in late diastole, E/e´ ratio between early mitral inflow velocity and early diastolic relaxation velocity, GLS global longitudinal strain, IVC inferior vena cava, IVSd interventricular septal thickness in diastole, LVEDVi left ventricular end-diastolic volume index, LVEF left ventricular ejection fraction, LVMI left ventricular mass index, mid myocardial, LVPWD left ventricular posterior wall dimensions, RVFWS right ventricular free wall longitudinal strain, TAPSE tricuspid annular plane systolic excursion, TR max PG pressure gradient from the peak tricuspid regurgitation velocity

**Table 3:** Multivariable linear regression models for subsegmental and segmental vessel fraction

	Total pulmonary vessel volume		Subsegmental vessel fraction		Segmental vessel fraction	
*Variable*	*Standardized Beta (± SD)*	*P-value*	*Standardized Beta (± SD)*	*P-value*	*Standardized Beta (± SD)*	*P-value*
LVMI	*-0.010*	*0.905*	-0.169	0.088	0.211	**0.032**
LVPWD	*0.214*	***0.005***	-0.163	0.056	0.215	**0.010**
IVSd	*0.059*	*0.485*	-0.255	**0.006**	0.291	**0.002**
LVEDVi	*0.183*	***0.008***	-0.080	0.279	0.082	0.268
LVEF	*-0.005*	*0.944*	-0.082	0.281	0.097	0.204
GLS	*-0.059*	*0.370*	-0.010	0.887	0.020	0.772
E/A	*-0.003*	*0.963*	0.012	0.865	0.040	0.560
E/e´	*0.001*	*0.988*	0.105	0.141	-0.085	0.235
IVC	*0.036*	*0.666*	-0.251	**0.006**	0.199	**0.028**
TAPSE	*0.055*	*0.397*	-0.143	**0.039**	0.112	0.108
RVFWS	*0.092*	*0.206*	-0.114	0.148	0.122	0.119
TR max PG	*0.082*	*0.320*	-0.160	0.070	0.189	**0.035**

*All models adjusted for age, sex, degree of emphysema, diabetes mellitus, hypertension, smoking, ischemic heart disease, time between CT scan and echocardiogram

E/A ratio of peak velocity in early diastole to peak velocity in late diastole, E/e´ ratio between early mitral inflow velocity and early diastolic relaxation velocity, GLS global longitudinal strain, IVC inferior vena cava, IVSd interventricular septal thickness in diastole, LVEDVi left ventricular end-diastolic volume index, LVEF left ventricular ejection fraction, LVMI left ventricular mass index, mid myocardial, LVPWD left ventricular posterior wall dimensions, RVFWS right ventricular free wall longitudinal strain, TAPSE tricuspid annular plane systolic excursion, TR max PG pressure gradient from the peak tricuspid regurgitation velocity

**Fig. 2:**
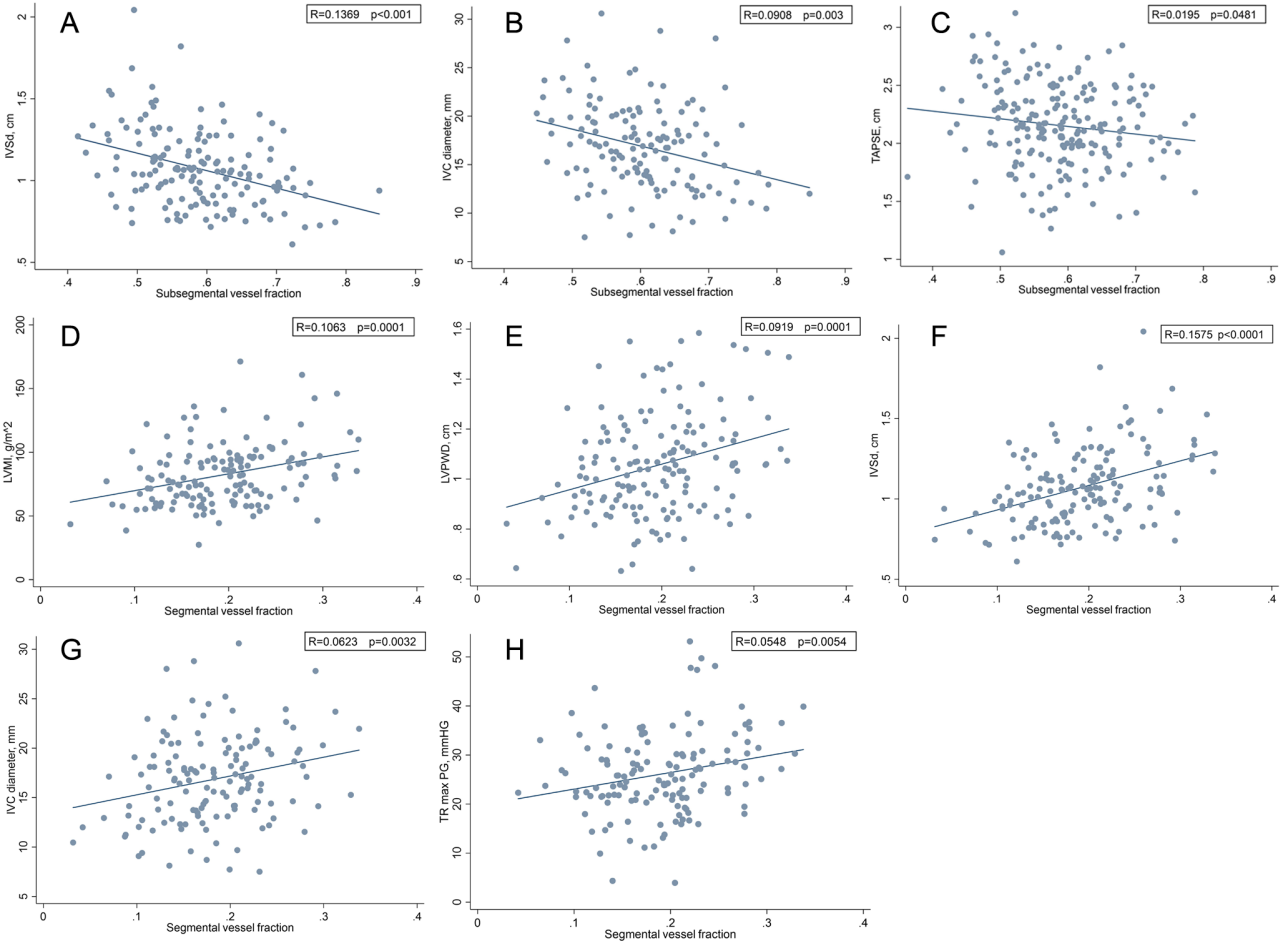
2A-D depicts the relationship between subsegmental vessel fraction and **(A)** IVSd **(B)** IVC diameter, **(C)** TAPSE, **(D)** LVMI. Figure 2E-H depicts the relationship between segmental vessel fraction and **(E)** LVPWD, **(F)** IVSd, **(G)** IVC diameter, **(H)** TR max PG **(A)** with subsegmental vessel fraction was negatively correlated **IVSd**, **(B)** subsegmental vessel fraction was negatively correlated with **IVC diameter**, **(C)** subsegmental vessel fraction was negatively correlated with **TAPSE**, **(D)** subsegmental vessel fraction was positively correlated with **LVMI**, **(E)** segmental vessel fraction was positively correlated with **LVPWD**, **(F)** segmental vessel fraction was positively correlated with **IVSd**, **(G)** segmental vessel fraction was positively correlated with **IVC diameter**, **(H)** segmental vessel fraction was positively correlated with **TR max PG**. IVC inferior vena cava, IVSd interventricular septal thickness in diastole LVEDVi left ventricular end-diastolic volume index LVMI left ventricular mass index LVPWD left ventricular posterior wall dimensions RVFWS right ventricular free wall longitudinal strain TAPSE tricuspid annular plane systolic excursion TR max PG tricuspid regurgitation tricuspid maximal pressure gradient

Unadjusted continuous correlations between the subsegmental vessel fraction and echocardiographic parameters are shown in Fig. 2a-h.

After multivariable adjustments, only LVPWD and LVEDVi remained significantly correlated with TPVV. While after multivariable adjustments, IVSd, IVC diameter, and TAPSE remained significantly correlated with subsegmental vessel fraction. After multivariable adjustments, LVMI, LPWD, IVSd, IVC, and TR max PG remained significantly correlated with segmental vessel fraction.

After adjustments, IVSd (standardized β=-0.257), and IVC (standardized β=-0.239) display the strongest correlations with subsegmental vessel fraction. While after adjustments, LVPWD (standardized β = 0.216) and IVSd (standardized β = 0.292) displayed the strongest correlations with segmental vessel fraction. Notably, LVEF was not correlated wither either subsegmental or segmental vessel fraction.

## Discussion

This study combines AI convolutional neural network techniques with measures of cardiac structure and function to demonstrate that the CT scan-measured subsegmental and segmental vasculature volume indexes correlate with echocardiographic measurements of cardiac structure and function in patients with COPD. This is the first study, to our knowledge, to specifically investigate this relationship in a cohort of well-characterized patients with COPD.

We found that patients with the lowest subsegmental vessel fraction i.e., more extensive pruning, had a higher extent of emphysema, had higher LVMI, due to thicker myocardial walls and smaller LV cavity size, higher IVC diameter, and higher LAVI, than patients with higher subsegmental vessel fraction. We found in the multivariable analysis that lower subsegmental vessel fraction was significantly linearly correlated with higher LV mass parameters, higher right atrial pressure, and impaired RV systolic function. In the multivariable analysis, we found segmental vessel fraction to be significantly correlated with higher LV mass parameters, higher right atrial pressure, and pulmonary pressure.

TAPSE was the only RV measure that showed a statistically significant correlation with subsegmental vessel fraction, suggesting that fewer subsegmental vessels with a CSA under 10 mm^2^ also have some consequence on the RV function and myocardial contractility. IVC diameter was found to be significantly negatively correlated with the subsegmental vessel fraction, suggesting that a lower amount of subsegmental vessels also could lead to a higher right atrial pressure.

The segmental vessel fraction was positively correlated with LVMI, LVPWD, and IVSd. Even after adjustment for clinical variables known to cause left-sided mass alteration, the measures remained significantly correlated, suggesting a higher blood volume in the larger vessels closer to the heart also impacts LV mass with a potential consequence of LV hypertrophy. TR max PG was the only RV measure significantly correlated with the segmental vessel fraction, suggesting that a lower number of segmental vessels could have a consequence of pulmonary vascular resistance and pulmonary pressure. Segmental vessel fraction was also significantly correlated with IVC diameter, suggesting that a lower number of segmental vessels also leads to a higher right atrial pressure.

The TPVV was positively associated with LVMI, LVPWD, IVSd and LVEDVi. However, after adjustments only LVPWD and LVEDVi remained significantly associated, suggesting that change in the total vascular volume in the lungs impacts both the LV mass and – volumes. Notably TPVV was the only vascular volume parameter associated with LV volume index, suggesting that the total vascular volume in the lung impacts the global LV diastolic performance. In general, oppositely directed correlations were found with subsegmental vessel fraction and segmental vessel fraction, which support the idea of large vessel dilatation as a compensatory mechanism to pruning of the small peripheral vessels. However, segmental vessel fraction was correlated to TR max PG while subsegmental vessel fraction was not. Subsegmental vessel fraction was correlated with TAPSE, while segmental vessel fraction was not. This suggests that the higher blood volume in the larger segmental vessels closer to the heart could have hemodynamic consequences on the RV and that segmental vessel fraction may be a more sensitive marker of higher pulmonary pressure. Furthermore, we found the subsegmental and segmental vessel fractions to be associated to with greater number of cardiac parameters compared to TPVV, which may suggest that the indexed vessel fractions are more sensitive markers of changes in cardiac structure and function.

Previous studies have primarily focused on small vessel fraction (small vessel volume/TPVV), regarding the severity of lung disease and less on cardiac impairment. Small vessels were defined as blood volume in vessels with nominal sizes below 5 mm^2^ in CSA. In these studies, a lower small vessel fraction was associated with the severity of airway disease, including impaired spirometry parameters, higher GOLD stage, higher risk of exacerbation, RV dysfunction, and higher all-cause mortality [2, 4, 7, 8].

A previous study by Wells et al. [2] investigated the association between small vessel fraction and cardiac function assessed by cardiac MRI in 24 patients with COPD. They found that small vessel fraction was significantly correlated with RV end-systolic volume index and RV mass index, and trending towards correlation with RVEF. We found TAPSE, a conventional echocardiographic surrogate of RVEF, to be significantly associated with the subsegmental vessel fraction. Both studies suggest that a lower number of small vessels in patients with COPD is associated with structural remodeling of the RV and impaired RV function. They did not find small vessel volume, nor the TPVV alone to be associated with any cardiac measures on MRI, suggesting that the indexed vessel fraction is a more sensitive marker of the lung-cardiac interplay than subsegmental vessel volume or TPVV alone. The vessel fraction being a more sensitive marker than the TPVV might be due to pruning causing a shift of blood from the smaller subsegmental vessel to the segmental vessels.

A study by Washko et al. [8] found similar results among previous and current smokers, where they found lower arterial volume to be independently associated with larger RV, with a 10 ml decrease in arterial small vessel volume giving a 1 ml increase in RV size measured with cardiac CT imaging. However, stratified multivariable models only found this to be true in patients with mild lung function impairment defined as an FEV1 above 73.6% of predicted. Furthermore, they found RV enlargement to be associated with a 63% higher risk of mortality, but only in patients with arterial pruning. No significant increased risk of death was seen without pruning [8]. Our study did not find any difference in FEV1 between the group of patients with the most extensive pruning and those with less extensive pruning. In addition, the GOLD stage did not correlate with the extent of pruning. This is probably due to the small number of patients in each GOLD stage group.

Another sub-study from the COPDgene study, by Rahaghi et al. [17] investigated the relations between small and large vessel fraction and RV function in 80 smokers using cardiac MRI. They found that both lower number of arterial and venous vessels were associated with dilation and hypertrophy of the RV with reductions in RV ejection fraction. They also found that an increase in arterial large volume fraction was associated with RV dysfunction. They suggested that the relationship between small vessel fraction and RV ejection fraction appeared to be driven by the arterial small vessel fraction [17]. They found that the arterial volume fraction remained of predictive power, after adjustment for the extent of emphysema. In our study, the extent of emphysema visible on CT scan as assessed by %LAA-950 was likewise minor, suggesting that microvascular destruction might be present before CT identified emphysema.

The existing literature suggests that LV systolic function is usually preserved in patients with COPD, whereas RV dysfunction is more common. However, like our study, a previous study [18] has found an association between pulmonary vessel volume and higher LV mass dimensions and lower LV filling. Aaron et al. [18] conducted a population-based study that investigated cardiac function with MRI and its association with TPVV. Reductions in total pulmonary vascular volume were associated with reduced left atrial volumes, reduced LV end-diastolic volume, and cardiac output, among previous and current smokers, including those without lung disease. They also found a lower TPVV to be associated with a greater LV mass/end-diastolic volume ratio, a parameter described as useful for the evaluation of LV concentric remodeling. Our study did not find any differences in the percentage of smokers (previous and current) in the group of patients with the most extensive pruning compared to those with less extensive pruning.

### Clinical perspective

These consistent radiographic results likely indicate that pulmonary vascular injury which occurs as a consequence of the development of COPD affects cardiac structure and function. CT scans may hold the potential of providing us with more information about cardiovascular risk amongst COPD patients, hence, it might be possible in the future to deduce which patients are at higher risk for cardiac impairment and therefore must be followed more closely or started in prophylactic treatment. Our study suggests that patients with more extensive pruning and greater compensatory dilation have more pathological cardiac remodeling and may potentially gain from further examination and closer follow-up.

Current techniques and measurements are based on images that are already acquired during a standard initial workup of COPD patients, making them easily implemented in clinical practice. The subsegmental vessel fraction along with other risk markers may help guide which patients may benefit from additional imaging e.g., echocardiography. This could potentially identify the patients who require further examination and closer follow-up.

### Limitations and strengths

Major strengths of our investigation are the prospective inclusion of patients in the cohort and the well-described patient group. In addition, this study includes the use of novel quantitative pulmonary vascular measures.

Our study is limited by its relatively small sample size and single-center experience, though we did demonstrate correlations between pulmonary vascular injury and cardiac structure and function in accordance with existing literature. In addition, patients did not have the echocardiographic examination and CT scans within a set period of time. Changes in cardiac function and anatomic parameters between the CT and echocardiogram may occur.

A considerable number of patients (n = 23) were excluded from the analysis due to inadequate computational vascular reconstruction which introduces the risk of selection bias. Although the exclusion was merely due to technical factors, additional studies with larger sample sizes are needed to define the complex relationships more definitively between CT measurements and cardiac dysfunction in patients with COPD.

## Conclusion

We found that pulmonary vasculature measurements — total pulmonary vascular volume, subsegmental vessel fraction, and segmental vessel fraction — were correlated with cardiac structural and functional measures in patients with COPD. Future use of AI on CT imaging holds the potential of providing us with more information about cardiovascular risk amongst COPD patients. In addition, it may serve as a tool for early identification of COPD patients in higher risk for cardiac impairment.

BMI body mass index, e´ early diastolic relaxation velocity, E/A ratio of peak velocity in early diastole to peak velocity in late diastole, E/e´ ratio between early mitral inflow velocity and early diastolic relaxation velocity, endo endocardial, epi epicardial, FEV1 forced expiratory volume in the first second, FEV1/FVC forced expiratory volume indexed to forced vital capacity, GLS global longitudinal strain, IVC inferior vena cava, IVSd interventricular septal thickness in diastole, LAVI left atrial volume index, LVEDVi left ventricular end-diastolic volume index, LVEF left ventricular ejection fraction, LVMI left ventricular mass index, mid myocardial, LVPWD left ventricular posterior wall dimensions, PA:A ratio pulmonary artery to aorta diameter ratio, RV FAC right ventricular fractional area change, RVFWS right ventricular free wall longitudinal strain, TAPSE tricuspid annular plane systolic excursion, TR max PG pressure gradient from the peak tricuspid regurgitation velocity. %LAA-950 = the percentage of low attenuation area less than − 950 HU (defined as emphysema).

## Data Availability

The data underlying this article is not publicly available due general data protection regulations but are available from the corresponding author on reasonable request.

## Abbreviations

AI: artificial intelligence
BV: blood volume
COPD: chronic obstructive pulmonary disease
CT: computed tomographic
IVC: inferior vena cava
IVSd: interventricular septal thickness in diastole
LVEDVi: left ventricular end-diastolic volume index
LVMI: left ventricular mass index
LVPWD: left ventricular posterior wall dimensions
RVFWS: right ventricular free wall longitudinal strain
TAPSE: tricuspid annular plane systolic excursion
TPVV: total pulmonary vascular volume
